# Drug Candidates for Autoimmune Diseases

**DOI:** 10.3390/ph15050503

**Published:** 2022-04-20

**Authors:** Sabrina Saurin, Myriam Meineck, Gerhard Erkel, Till Opatz, Julia Weinmann-Menke, Andrea Pautz

**Affiliations:** 1 1st Department of Medicine, University Medical Center of the Johannes Gutenberg University, 55131 Mainz, Germany; sabrina.saurin@unimedizin-mainz.de (S.S.); myriam.meineck@unimedizin-mainz.de (M.M.); 2 Research Center for Immunotherapy (FZI), University Medical Center of the Johannes Gutenberg University, 55131 Mainz, Germany; 3 Department of Molecular Biotechnology and Systems Biology, Technical University, 67663 Kaiserslautern, Germany; erkel@bio.uni-kl.de; 4 Department of Chemistry, Johannes Gutenberg University, 55099 Mainz, Germany; opatz@uni-mainz.de; 5 Department of Pharmacology, University Medical Center of the Johannes Gutenberg University, 55131 Mainz, Germany

**Keywords:** autoimmunity, inflammation, natural products, macrolactone

## Abstract

Most of the immunosuppressive drugs used in the clinic to prevent organ rejection or to treat autoimmune disorders were originally isolated from fungi or bacteria. Therefore, in addition to plants, these are valuable sources for identification of new potent drugs. Many side effects of established drugs limit their usage and make the identification of new immunosuppressants necessary. In this review, we present a comprehensive overview of natural products with potent anti-inflammatory activities that have been tested successfully in different models of chronic inflammatory autoimmune diseases. Some of these candidates already have passed first clinical trials. The anti-inflammatory potency of these natural products was often comparable to those of established drugs, and they could be used at least in addition to standard therapy to reduce their dose to minimize unwanted side effects. A frequent mode of action is the inhibition of classical inflammatory signaling pathways, such as NF-κB, in combination with downregulation of oxidative stress. A drawback for the therapeutic use of those natural products is their moderate bioavailability, which can be optimized by chemical modifications and, in addition, further safety studies are necessary. Altogether, very interesting candidate compounds exist which have the potential to serve as starting points for the development of new immunosuppressive drugs.

## 1. Introduction

Loss of self-tolerance of the immune system to the body´s own tissue leads to autoimmune diseases. Aberrant immune-mediated responses by leukocytes and/or antibodies result in pathological changes and dysfunctions of organs, such as kidneys, joints, skin, heart, lung, or the brain. A distinction can be made between organ- or tissue-specific (e.g., type-1 diabetes mellitus and multiple sclerosis (MS)) and systemic (e.g., systemic lupus erythematosus (SLE) and Sjögren´s syndrome) disease. In general, there is a prevalence of autoimmune diseases in females [1]. Triggers are usually multifactorial and can depend on genetics, environment, hormonal status, and immunological effects [2].

The current treatment strategies of various autoimmune diseases are based on immunosuppressive therapies, depending on the severity and type of the manifestations. General standard therapies include the use of glucocorticoids, which are then supplemented with other immunosuppressive agents to reduce the dosing of glucocorticoids. These immunosuppressants include cyclosporine, mycophenolate mofetil, cyclophosphamide, etc. Unfortunately, all these medications have a variety of undesirable side effects (e.g., nephrotoxicity), which is particularly problematic since they often have to be used in very young patients. Although cyclosporine is well known for its nephrotoxicity, mycophenolate mofetil and cyclophosphamide can be used in treating autoimmune diseases with kidney involvement and renal insufficiency, such as lupus nephritis. Notably, the prescription of these immunosuppressants is associated with increased occurrences of infections.

Therefore, one of the most important goals in the therapy of autoimmune diseases is to find new therapeutic approaches with a reduced side effect profile. One step in this direction is targeted antibody therapies, such as TNF-alpha inhibitors (infliximab, adalimumab, etc.), or B-cell-suppressing therapies, such as rituximab or belimumab [3,4,5,6].

Many compounds used in the clinic to treat autoimmune disorders or to prevent organ rejection after transplantation, such as cyclosporin (a cyclopeptide from the ascomycete fungi *Tolypocladium infatum* and *Clindrocarpon lucidum*), tacrolimus/FK506 (macrolide lactone from the soil bacterium *Streptomyces tsukubaensis*), sirolimus/rapamycin (from *Streptomyces hygroscopicus*), and everolimus (a macrocyclic lactone isolated from *Streptomyces hygroscopicus*), are natural products originally isolated from fungi and bacteria. This also applies to fingolimod, an immunosuppressant used in the treatment of MS, which is a chemical-modified product of myriocin, a metabolite of the fungus *Isaria sinclairii* [7]. Moreover, in traditional medicine, such as traditional Chinese medicine, plants or plant extracts also have long been used as herbal medicines. 

New natural products with anti-inflammatory or immunomodulatory effects continue to be discovered in plants or by screening of secondary metabolites from fungi, herbs, and microorganisms. We summarized in this review the current knowledge regarding natural products which may be promising drug candidates for the treatment of inflammation and autoimmune diseases. Therefore, we focused on natural products, which were investigated in early clinical trials, in vivo or in vitro models of chronic inflammatory autoimmune diseases, predominantly in rheumatoid arthritis (RA) or SLE. We decided not to include phytochemicals that were studied in in vitro models only because here it is hard to judge regarding the potential of those substances due to the limited amount of data.

## 2. Natural Products in Clinical Trials for Autoimmune Diseases

Some natural products are already being used in clinical trials (Table 1). They are either administered as drug alone or as an adjuvant to standard therapy. In the first part, we will summarize the most important information about various natural products already tested in clinical trials for different autoimmune diseases.

### 2.1. Curcumin

Curcumin is an ingredient of turmeric, a culinary spice traditionally used in India, China, Africa, and Jamaica. Turmeric is obtained from the rhizomatous herbaceous plant *Curcuma longa* and contains 2.5–6% curcuminoids, consisting of curcumin, demethoxycurcumin, and bisdemethoxycurcumin [43]. Curcuminoids were used in traditional herbal medicine throughout history and also show therapeutic effects in modern medicine as a potent anti-inflammatory, anticancer, antimicrobial, and neuroprotective agent in clinical trials [44,45,46].

In many studies, curcumin is described as a safe and nontoxic compound [47,48], unfortunately with a low bioavailability resulting from poor solubility, low absorption, rapid metabolism, and excretion [49,50]. Strategies to improve the bioavailability of curcumin resulted in the development of nanoscopic curcumin formulations (“nano-curcumin”) [51] and Pickering emulsions [52].

Curcumin has many molecular targets and affects different signaling pathways. The anti-inflammatory effect of curcumin is attributed to the suppression of a variety of cell signaling pathways, including nuclear factor κB (NF-κB), signal transducer and activator of transcription 3 (STAT3), nuclear factor erythroid 2-related factor 2 (Nrf-2), reactive oxygen species (ROS), and cyclooxygenase-2 (COX-2) [47]. A curcumin-dependent downregulation of the chemokines C-X-C motif chemokine ligand (CXCL)-1 and CXCL-2 in cancer cells was observed, which was influenced by NF-κB, a transcription factor associated with inflammation and cancer progression [9]. Tan et al. observed a reduction in interleukin (IL)-1β, IL-6, tumor necrosis factor α (TNF-α), and monocyte chemoattractant protein-1 (MCP-1) in curcumin-treated lipopolysaccharide (LPS)-stimulated RAW 264.7 cells, a murine macrophage cell line. Similar results were obtained in a cisplatin-induced acute kidney injury mouse model. Inhibition of NF-κB activation [8] by blocking inhibitor of NF-κB (IκB)-kinase and protein kinase B activities [53] seems to be the mode of action of curcumin. In addition, it was shown that curcumin reduced oxidative stress by inducing the important ROS scavenging enzyme glutathione-S-transferase [54] and activated heme oxygenase-1 (HO-1) via the Nrf-2/antioxidant responsive element (ARE) pathway [55].

In vivo studies of curcumin treatment in experimental autoimmune disorders show an improvement of the disease [10,11,12]. One example is the study from Dent et al. [10] in NZBWF1 mice, an established experimental model of SLE. After two weeks of oral treatment with curcumin (500 mg/kg/day), they observed decreased autoimmune activity, less renal injury, and improved renal function in the treated SLE mice compared to the untreated group. Based in part on the statistically significant reduction in blood urea nitrogen (BUN) level and autoantibodies as well as lower glomerulosclerosis in curcumin-treated SLE mice, the authors propose curcumin as a good complementary treatment option [10].

Furthermore, curcumin has been used in many clinical trials in autoimmune disease. In a randomized and single-blinded pilot study, they tested oral administration of curcumin (500 mg), diclofenac sodium (50 mg), or the combination of both in 45 patients with RA. The treatment given twice a day for 8 weeks resulted in a significant reduction in clinical arthritis activity in all three groups. The highest reduction in subjective pain score (visual analogue scale (VAS)) was observed in the curcumin-treated group and only curcumin-treated patients had a statistically significant change in the inflammation marker C-reactive protein (CRP) in serum. Moreover, curcumin was described as generally safe and well-tolerated [13]. Another randomized, double-blinded, and placebo-controlled study with RA patients using 250 or 500 mg curcumin twice daily for 90 days resulted in improvement of different clinical marker (erythrocyte sedimentation rate (ESR), CRP) and disease activity scores (VAS, disease activity score 28 (DAS28), American college of Rheumatology score (ACR)) compared to the placebo group. These improvements were already observed at the low dose (250 mg) of curcumin; both doses were well-tolerated and without any side effects [14]. A nano-curcumin preparation shows a better bioavailability and was tested in a clinical trial with 57 patients with oral lichen planus (OLP). OLP is an autoimmune disease of mucous membranes and can lead to painful lesions. In the double-blinded, randomized phase 3 study, they treated the patients with curcumin (80 mg/day) compared to prednisolone (10 mg/day) as a control. The results showed a decreased level of pain and OLP lesions in both groups, without significant difference between them, indicating that nano-curcumin may be used as an alternative treatment for OLP lesions [15].

### 2.2. Resveratrol

Resveratrol is a polyphenolic phytoalexin that was first isolated in 1939 from the root of *Veratrum grandiflorum*, the white hellebore, and was also found in many different edible parts of plants, such as berries, red grapes, peanuts, pistachios, and plums [56].

Studies and clinical trials to test the beneficial effects of resveratrol were performed in various diseases, such as diabetes mellitus, obesity, cancer, and in neuronal, cardiovascular, kidney, and inflammatory diseases [57]. The poor bioavailability of the compound limited its clinical use, but a formulation of micronized resveratrol (SRT501) seems to have a higher bioavailability [58] and could be an alternative to the natural product.

Resveratrol downregulated proinflammatory cytokines and chemokines, such as TNF-α, C-C motif ligand 3 (CCL3), IL-1β, IL-6, and MCP-1, in animals as in human diseases, such as diabetes [17,59], and reduced the expression of the inflammatory and oxidative stress marker CRP, c-Jun N-terminal kinase 1 (JNK-1), IκB kinase β (IKK-β), neutrophil cytosol factor 1 (p47phox), and toll-like receptor 4 (TLR4) in cells from healthy donors [60]. Many studies postulated that sirtuin-1 (SIRT1) and AMP-activated protein kinase (AMPK) are two of the crucial targets activated by resveratrol [61,62], which modify the activity of other transcription factors, such as NF-κB [63], and enzymes, such as phosphodiesterases [64,65], directly or indirectly. In addition, resveratrol inhibited COX 1 and 2 directly by regulation of the transcription. Thus, resveratrol inhibited COX-2 expression via downregulation of protein kinase B (Akt), mitogen-activated protein kinase (MAPK), and NF-κB by blocking IκB kinase activity in vivo [66,67]. In addition, resveratrol reduces mRNA expression of proinflammatory mediators on the post-transcriptional level by enhancing the activity of the mRNA-destabilizing protein KSRP [68]. Furthermore, the cardiovascular protective properties of resveratrol are well known. Through antioxidative effects and the improvement of endothelial nitric oxide synthase (eNOS) activity, resveratrol improves vascular function and reduces hypertension [69].

In cardiac myosin-immunized rats, an experimental model of autoimmune myocarditis, Yoshida et al. observed a significant ameliorated myocardial injury and better cardiac function in resveratrol-treated rats compared to the control group. They measured after 14 or 21 days of treatment with resveratrol (50 mg/kg i.p. per day) an attenuated heart weight, a decreased cellular infiltration, as well as lower fibrosis in the myocardium. In addition, they observed a decrease in inflammation markers, such as TNF-α and inducible nitric oxide synthase (iNOS), in the treated rats [16]. In another study, the effect of oral administration of resveratrol (200 or 400 mg/kg) in RA was investigated in bovine type-II collagen (BIIC)-induced rats. The treatment downregulated the expression of the proinflammatory cytokines IL-1β, IL-6, MCP-1, and TNF-α in serum, and ameliorated synovitis and RA-related pathological hallmarks, such as inflammatory cell infiltration in synovial tissue. Moreover, they presented evidence for inhibition of the MAPK signaling pathway, most likely due to reduced ROS accumulation in vitro [17]. In the pristine-induced SLE mouse model, the oral treatment with resveratrol (25 or 50 mg/kg) in combination with the bio-enhancer piperine (1/10 of the dose of resveratrol, to improve its bioavailability) over 4 months ameliorated lupus-associated manifestation in kidneys, liver, and lungs. The lupus-induced increase in interferon α (IFN-α), IL-6, and TNF-α levels was improved by resveratrol, as well as the renal manifestation, such as proteinuria. At the same time, resveratrol showed no effect on autoantibody formation and no or only a small effect on mortality in SLE mice [18].

A clinical trial with resveratrol as an adjuvant therapy was performed as a randomized and controlled trial on 100 RA patients. Resveratrol was applied at a dose of 1 g per day over 3 months in addition to the conventional treatment. A reduction in biochemical markers CRP, ESR, undercarboxylated osteocalcin, matrix metalloproteinase-3 (MMP-3), TNF-α, and IL-6 was observed in the resveratrol-treated patients. In addition, the clinical scores of swollen 28-joint count (SJC-28), tender 28-joint count (TJC-28), DAS28, and ESR were also significantly reduced compared to the control group. The results indicate that resveratrol is a suitable treatment option for RA patients [19].

### 2.3. Quercetin

Quercetin is a highly abundant flavonoid found in many fruits, leaves, and flowers. The average consumption of this flavonoid is around 25–50 milligram per day [70]. Normally, the bioavailability of quercetin is low, but enzymatic modifications in the intestine and colon have been shown to generate bioavailable quercetin metabolites [71,72].

Many reports describe potent anti-inflammatory and antioxidative effects of quercetin. The inhibition of p38 MAPK, extracellular signal-related kinases (ERK1/2), and NF-κB led to the reduction in cytokine (e.g., TNF-α, IL-6, and IL-1), COX, iNOS, and lipoxygenase expression in different cellular models, such as LPS-activated human U937 cells or murine RAW 264.7 macrophages. The anti-inflammatory effect was accompanied by efficient reduction in oxidative stress, mediated by the modulation of signal transduction pathways, such as HO-1/Nrf2, ROS reduction, and improved glutathione function [73].

In vivo studies in different mouse arthritis models demonstrated beneficial effects mediated by quercetin. Clinical arthritis symptoms were reduced, most probably due to quercetin-mediated downregulation of proinflammatory cytokine and chemokine expression (e.g., TNF-α, IFN-γ, MCP1, IL-6, or IL-17) [20,21,22]. In two different lupus mouse models, quercetin treatment improved glomerulonephritis, one of the most severe complications in lupus disease. As underlying mechanisms, impaired activation of CD4^+^ T cells and macrophages [23], reduced expression of TNF-α, IL-6, and transforming growth factor β (TGF-β), and decreased oxidative stress [24] were discussed. The putative therapeutic effects of quercetin were examined in a clinical trial (randomized, double-blind, placebo-controlled), in which 50 RA patients were treated with quercetin (500 mg/day) or placebo for 8 weeks. The authors detected reduced clinical arthritis activity monitored by reduced DAS28 and health assessment questionnaire (HAQ) scores in the quercetin-treated patients. This was accompanied by reduced serum plasma levels of TNF-α. No significant toxic side effects were recognized [25].

### 2.4. Sinomenine (SIN)

The alkaloid sinomenine (SIN) is the main active ingredient in the roots and stems of the plant *Sinomenium acutum*. It has been used for a long time in traditional Chinese medicine to treat RA and, meanwhile, it has been approved by the Chinese government for RA therapy [26]. Most effective seems to be an SIN hydrochloride formulation, which allows a sustained drug release and absorption [74].

The anti-inflammatory properties of SIN were demonstrated in LPS-induced RAW 264.7 macrophages and a collagen-induced arthritis (CIA) mouse model. In vitro and in vivo, SIN treatment reduced secretion of RA-associated cytokines, such as IL-6, granulocyte-macrophage colony-stimulating factor (GM-CSF), IL-12p40, TNF-α, IL-1β, CXCL1, CCL5, or MCP-1 [26]. In macrophages, a putative mode of action could be inhibition of the activated TLR4/NF-κB signaling pathway [75]. SIN treatment suppressed the NF-κB pathway in human monocyte-derived dendritic cells [27] and reduced iNOS expression via inhibiting T-box transcription factor (T-bet)/IFN-γ pathway in an experimental rat model of autoimmune encephalomyelitis [28]. SIN also inhibited fibrosis via regulation of the Janus kinases (JAK) 2/STAT3/suppressor of cytokine signaling 1 (SOCS1) pathway in streptozocin-induced diabetic rats [76]. The drug was tested in at least two different randomized controlled clinical trials. One 24-week open-label study with 120 patients compared the effect of SIN (120 mg/day) + methotrexate (MTX) with the well-established combination therapy of MTX + leflunomide (20 mg/day). In both study arms, a similar improvement in RA disease activity was determined according to the ACR criteria, but, in the SIN + MTX group, less adverse effects, such as gastro-intestinal problems or liver toxicity, occurred [29]. In the second clinical trial, 25 RA patients received SIN (60–120 mg) two times a day and, in the control group, 24 RA patients were treated with MTX (7.5–10 mg) and folic acid tablets (5 mg) orally once a week for 3 months. SIN treatment reduced production of proinflammatory cytokines and DAS28 in RA patients to a similar degree as MTX treatment [26].

### 2.5. Baicalein (BE)/Baicalin (BA)

Baicalein (BE) and Baicalin (BA) are two flavonoids isolated from the roots of the plant *Scutellariae radix* known as *Sucullaria baicalensis* [77]. This herb is used in traditional Chinese medicine for the treatment of diarrhea, fever, cough, and jaundice.

In clinical studies, BE and BA are described to be safe and well-tolerated [77,78]. Both have a moderate bioavailability. BA shows a moderate absorption in the stomach and a poor absorption in the colon, while BE has better bioavailability in both. Therefore, after oral administration, there is more BE in the blood [30]. Several formulations, such as micelles, nanocrystals, and gels, were developed to improve the bioavailability of BE and BA [79,80].

Both substances exhibit antioxidative properties, yet the effect of BA appears to be mainly based on scavenging superoxide, while BE inhibits xanthine oxidase [81,82]. Moreover, BA has an anti-inflammatory effect in vivo by inhibiting NF-κB activation and p38 MAPK phosphorylation [83]. BA also reduced the expression of TLR2 and TLR4, so that the TLR2/4 signaling pathway is inhibited [84,85]. In addition, BA and BE have been shown to inhibit the JAK/STAT pathway [86]. Xu et al. demonstrated that BE and BA have selective therapeutic effects on autoimmune diseases in different experimental models by regulation of cell proliferation and STAT gene expression. Despite the similar structure, both substances act on different cell types [30].

BA and BE were tested in different autoimmune models in vivo. Xu et al. tested both substances in the RA model of CIA and in the dextran sodium sulfate (DSS)-induced colitis model. The severity of CIA was attenuated by BE (20 mg/kg) treatment through suppression of T-cell proliferation. The DSS-induced colitis improved under the treatment with BA (20 mg/kg). Here, BA primarily acted on colon epithelial cells [30]. In another study with experimental colitis in rats, BA downregulated proinflammatory mediators, such as MCP-1, COX-2, TNF-α, IL-1β, and IL-6, in serum and in LPS-stimulated RAW 264.7 cells [31]. In the experimental autoimmune encephalomyelitis (EAE) mouse model of MS, the treatment with 100 mg/kg per day of BA reduced cytokine (e.g., IL-17, IFN-γ, IL-1β, and IL-6) and chemokine expression (e.g., CXCL1, CXCL2, and CXCL8) in the central nervous system. The Th1 and Th17 cells were selectively suppressed via STAT and NF-κB signaling pathway in BA-treated EAE mice [32].

In a randomized, double-blinded, and placebo-controlled clinical trial, BA was tested on coronary artery disease and RA in 374 patients. They received a daily dose of 500 mg BA or placebo for 12 weeks and a significantly improved lipid profile in the BA-treated patients was observed. Furthermore, significantly more patients with BA treatment have a good or moderate response according to the European League against Rheumatism (EULAR) score compared to the placebo group [33].

### 2.6. Paeoniflorin (PAE)

Paeoniflorin (PAE) is a monoterpene glucoside isolated from *Oaenonia lacriflore*, the Chinese peony. The glycoside of peony (TGP) is used in traditional Chinese medicine to treat pain, inflammation, and immune disorders [87]. The major active compound of TGP is PAE [88]. TGP was approved by the China Food and Drug Administration for the treatment of RA in 1998 [89], and is also used in other autoimmune diseases, such as SLE [90] or Sjögren syndrome [91], without significant side effects [92]. Despite the biological effectiveness of TGP, the aglycon PAE only has a poor oral bioavailability, but there are new methods to enhance the bioavailability, e.g., through micellar formulations [93].

In various inflammatory and autoimmune animal models, treatment with PAE has a good efficacy [94,95,96,97,98,99]. Among others, PAE regulates the activity and function of immune cells; for example, it inhibits B lymphocyte activation, proliferation, and differentiation [100] and suppresses M1 macrophage cell activity. Moreover, PAE enhanced M2 cell function via increasing IL-4/STAT6 signaling pathway [101,102]. In in vivo studies, PAE also regulates signaling pathways mediated by cytokines. In two RA rat models, PAE showed an anti-inflammatory effect through suppressing MAPK/NF-κB signaling pathway [34] and suppressed TGF-β-mediated epithelial–mesenchymal transition (EMT) and tissue fibrosis, as well as inflammation via TGF-β/small mother against decapentaplegic (Smad) signaling pathway in two other models [103,104]. In addition, PAE inhibited immune cells via JAK/STAT3 pathway [105] and hindered the proliferation, migration, and inflammation of RA-fibroblast-like synoviocytes (FLSs) by affecting the FAM120A/miR-671-5p/MDM4 pathway [35]. PAE was also tested in a double-blind, randomized clinical trial in RA patients. In this study, treatment with PAE plus cucumis polypeptide injections (CCPI) and treatment with disease-modifying antirheumatic drugs (DMARDs), such as methotrexate and leflunomide, were compared. One study outcome was that the maximum improvement was significantly higher in the DMARDs group. After 6 months, the difference in the American College of Rheumatology 20% improvement response criteria (ACR20) was not significant between the two groups. However, the PAE-and-CCPI-treated group showed fewer side effect compared to the DMARDs group. The authors concluded that PAE plus CCPI is an alternative for patients who cannot use DMARDs and it may be a safer option for long-term treatment of RA patients when DMARD toxicity is an issue [36].

### 2.7. Hesperidin (HES)

The flavonoid glycoside hesperidin (HES) was isolated first from citrus peel and also later found in bergamot fruit, banana fruit, lemon fruit, and lemon peel. Some studies demonstrated a positive effect from HES in hypertension [106] and diabetes [107]. Moreover, HES showed anti-inflammatory, antioxidant [37], and neuroprotective effects [108].

The bioavailability of HES is limited, but a double-blinded randomized crossover study with 16 healthy volunteers demonstrated that the removal of the rhamnose (monosaccharide) unit from the disaccharide glycone of HES to form herperetin-7-glucoside improves the bioavailability relative to native HES [109].

HES inhibited COX2 gene expression in LPS-induced RAW 264.7 cells [37] and suppressed the expression of the inflammatory cytokines IL-8, TNF-α, IL-1β, IL-6, and IL-12 in vitro and in vivo by the inhibition of IκB and MAPK signaling pathway [110]. Moreover, HES showed anti-inflammatory effects in various experimental models of autoimmune and inflammatory diseases. HES treatment in an ovalbumin (OVA)-induced asthma mouse model reduced IL-4, IL-5, IL-13, and IL-17 production, as well as OVA-specific IgE expression by inhibition of the GATA binding protein 3 (GATA3) transcription factor to the DNA. In addition, HES treatment reduced the infiltration of inflammatory cells into the bronchoalveolar lavage fluid (BALF) [38,39]. In the antigen-induced arthritis (AIA) mouse model, treatment with 20 mg/kg HES per day ameliorated the AIA and reduced the protein level of MMP-3, MMP-9, and MMP-13 in fibroblast synovial cells. Furthermore, HES inhibited the polarization of macrophages to M1 macrophages in this model [40]. HES (80 or 160 mg/kg) inhibited secondary paw swelling also in adjuvant arthritis (AA) in rats and decreased the transcription and production of TNF-α, IL-10, and IL-1β from synoviocytes. At the same time, HES suppressed the proliferation of synoviocytes in AA rats [41].

One review summarized the anti-inflammatory properties of orange juice, including flavonoids such as HES and naringenin, in human studies [111]. Another double-blind, randomized, and placebo-controlled clinical trial investigated the effect of 3 g alpha-glucosylhesperidin (HES-G) per day in 19 RA patients additional to the standard therapy. To verify the effectiveness, the ACR score was used. By 33% of the HES-G group and only by 10% of the placebo group an ACR20 improvement was detected. No improvement in serum CRP was observed in the HES-G-treated and placebo-treated group. HES-G is described as a safe and effective short-term treatment in RA patients, evaluated by ACR20, and is maybe useful as a complementary medicine in RA [42].

## 3. Natural Products in Animal Research for Autoimmune Diseases

Other natural products that have not yet been used in clinical trials, but in in vitro and in vivo studies on various experimental models of autoimmune diseases are displayed in the following part (see also Table 2). Some of these compounds have been already tested in clinical trials of nonautoimmune diseases or used in other clinical indications. Therefore, in this part, we have compiled the most important information for many of these substances. For RA, an overview of plant secondary metabolites investigated in diverse models has been authored by Santiago et al., which also include some less intensively studied ones [112].

### 3.1. Celastrol

Celastrol is a triterpenoid and was first isolated from the plant *Tripterygium wilfordii* Hook F. (TWHF) in 1926. TWHF is used in traditional Chinese medicine for the treatment of inflammatory and autoimmune disorders, such as arthritis [139]. It is also used in clinical practice in China and has been developed for the treatment of RA since the 1970s [139,140]. Celastrol also has anticancer [141], anti-inflammatory [142,143], and antioxidant activities [143,144].

The anti-inflammatory effect of celastrol in liver fibrosis can be attributed to activation of the AMPK/SIRT3 signaling pathway in vitro and in vivo [142]. Celastrol downregulated the expression of p38MAPK and NF-κB p65 in a diabetes rat model [145] and blocked the activation of STAT3 in rats with AA, a model of RA [113]. Moreover, celastrol treatment ameliorated cisplatin-induced nephrotoxicity by suppressing NF-κB in mice [146] and also had effects in cancer models by activating ROS/JNK signaling and blocking Akt/mammalian target of rapamycin (m-TOR) signaling pathway [147].

In the AIA rat model of arthritis, celastrol treatment (3 g/kg/day) suppressed the expression of the proinflammatory cytokines IL-6, IL-17, and IFN-γ, reduced MMP-9 activity, and reduced serum levels of anticyclic citrullinated peptide (aCCP) antibodies [114]. Furthermore, celastrol downregulated the expression of cytokines and chemokines, such as IL-8, IL-19, IL-23, CCL5, CCL20, and CXCL1, and inhibited the T cell differentiation from Th17 and Th22 in a 2D and 3D human in vitro model of psoriasis. [115]. There are no clinical trials in humans with autoimmune diseases.

### 3.2. Glycyrrhizin (GLY)

Licorice originates from the plant *Glycyrrhiza glabra*, and parts as well as extracts of this plant are used in pharmaceutical and food industries [148]. Furthermore, it is used in traditional Chinese medicine and folk medicine for gastrointestinal problems, bronchitis, arthritis, cough, respiratory infections, and tremors. The primary active component of licorice is glycyrrhizin (GLY), a triterpenoid saponin.

The oral bioavailability of GLY is low, but the intestinal microflora metabolized it to glycyrrhetic acid and monoglucuronyl glycyrrhetic acid, and both can be easily absorbed by the gastrointestinal tract [149].

GLY suppressed inflammation and cell apoptosis in vitro and in vivo by inhibiting the expression of the high-mobility group box-1 gene (HMGB1), coding for a nuclear protein that is involved in inflammatory processes [150], via p38/p-JNK signaling pathways [151,152]. Moreover, GLY could reduce oxidative stress by inhibiting MAPK and NF-κB pathways and activating AMPK/Nrf2 signaling [153]. Treatment with GLY in CIA rats showed a significant reduction in HMGB1, beclin-1, and light chain 3 (LC-3) proteins, as well as an induction of Nrf2-DNA-binding activities. Overall, an improvement of arthritis in CIA rats could be observed [116]. So far, human trials were performed in atopic dermatitis with licorice extract [154], in chronic hepatitis C with GLY [155] and with GLY acid, in addition to selective serotonin reuptake inhibitors (SSRI) in the treatment of depression through an anti-inflammatory approach [156]. No clinical trials in autoimmune disorders have been performed so far.

### 3.3. Artemisinin-Type Drugs

Artemisinin (ART) was isolated from *Artemisia annua* and is used in traditional Chinese medicine for the treatment of fever. In clinical practice, ART is used as anti-malaria drug. Tu Youyou was awarded the Nobel Prize in Medicine in 2015 for her work on malaria and the isolation of ART.

Treatment with ART showed an immunomodulatory effect via induction of forkhead box P3 (FoxP3) protein in CIA mice [117]. The treatment also inhibited the phosphorylation of JAK-2 and STAT3 in the SLE model of MRL/lpr mice [118] and blocked STAT1 phosphorylation in vitro [119]. Moreover, ART suppressed the activation of TLR4 and NF-κB and activated Nrf2 signaling pathway in vivo [120,157].

ART is being tested in different disease models, including autoimmune diseases. In CIA rats, a model of RA, ART (5–20 mg/kg/day) increased expression of FoxP3 and decreased IL-17 expression in CD4^+^ T lymphocytes. This reverses the imbalance of Th17 and Treg cells associated with RA [117]. The oral application of ART in MRL/lpr mice, an established model of SLE, prolonged the survival, ameliorated lupus nephritis symptoms, and decreased the level of anti-dsDNA antibody deposition in the kidneys of SLE mice [118]. In human umbilical vein endothelial cells (HUVECs) and peripheral blood mononuclear cells (PBMCs) from SLE patients, ART decreased macrophage migration inhibitory factor (MIF), a key regulator of atherosclerosis in SLE, by reduction in IFN-α overexpression via inhibition of STAT1 phosphorylation in vitro [119].

The ART metabolite dihydroartemisinin (DHA) decreased TLR4 expression, inhibited phosphorylation of interferon regulatory factor 3 (IRF3), and downregulated the release of type 1 interferons (IFN-α and IFN-β) in vitro [120]. Treatment with 100 mg/kg DHA and/or 5 mg/kg prednisone every day for 2 months in SLE mice reduced antinuclear antibody (ANA) production, anti-dsDNA antibody and anti-snRNP/Sm antibody levels, creatinine and BUN in serum as well as in urine. Furthermore, it decreased the infiltration of inflammatory cells in kidney, and DHA shows an immunomodulatory effect on Treg/Th17 in SLE mice [121]. DHA (50–75 mg/kg) also reduced inflammation by a reduction in cytokines, such as TNF-α, IL-1β, IL-6, and IL-10, in different mice models of asthma and acute lung injury [122,123].

ART is also being tested in other experimental diseases, including neurological diseases (general neuroinflammation, autoimmune encephalitis, Alzheimer´s diseases), skin diseases (dermatitis, rosacea, psoriasis), inflammatory bowel disease, nephritis, type 1 diabetes mellitus, and atherosclerosis [158].

### 3.4. Sophocarpine (SPC)

The alkaloid sophocarpine (SPC), isolated from the herb *Sophorae subprostratae* is used in traditional Chinese medicine and has immune-regulatory, antiviral, and antitumor properties [159,160,161].

SPC suppressed inflammatory response in vitro and in vivo by suppressing MAPK and NF-κB signaling [124] and attenuated liver fibrosis by inhibiting the TLR4 pathway [162]. Moreover, SPC inhibited STAT1 activation and overexpression of suppressor of cytokine signaling 1 (SOCS1), resulting in a downregulation of T-bet, inhibiting the activation of Th1 cells and expression of IFN-γ in vivo [163].

SPC exhibited a low cytotoxicity in LPS-induced FLSs and downregulated the LPS-induced proinflammatory cytokines TNF-α, IL-1β, IL-6, and IL-12 on mRNA and protein levels. Furthermore, SPC reduced clinical symptoms and cytokine level (TNF-α, IL-1β, IL-6, and IL-12) in serum of CIA mice, a model of RA [124]. In MRL/lpr mice, a model of SLE, SPC treatment (100 mg/kg) caused a reduction in cytokines (TNF-α, IL-1β, IL-6) in serum and kidneys. In addition, the treatment reduced proteinuria, anti-dsDNA antibody, and immune complex deposition in the kidneys, as well as decreasing the protein levels of NLR family pyrin domain containing 3 (NLRP3), inflammasome adaptor molecule ASC, caspase-1, and IL-1β in the kidney. SPC treatment also suppressed the NF-κB activation, possibly by inhibiting IKK [125]. SPC-treated OVA-induced asthma mice showed a reduction in IgE in serum and an inhibition of inflammatory cell infiltration. Moreover, SPC treatment improved lung tissue pathology, reduced the cytokine production in BALF (IL-4, IL-5, and IFN-γ), and regulated Th1/Th2 immune imbalance in an asthma model [126].

Only one clinical trial (randomized, double-blind, and placebo-controlled) was performed with *Sophora alopecuroides var. alopecuroides* (shrub) seed extract for opioid detoxification. This study shows a good safety and tolerability of the extract [164].

### 3.5. Berberine (BBR)

Berberine (BBR), a protoberberine alkaloid, is found in roots, rhizomes, stem, and bark of the plant *Berberis vulgaris*, but also in many other Berberis, Coptis, and Hydastis species. In herbal medicine, Berberis has been used to treat dysentery, diarrhea, stomatitis, and hepatitis [165,166,167,168]. The alkaloid BBR has been described as a regulator of lipid and glucose metabolism [169], suppressor of tumor cell proliferation [170], and inducer of apoptosis [171].

Although the poor oral bioavailability of BBR limits its clinical use [172,173], BBR is used in current medicine as nonprescription drug for diarrhea [174] and is well-tolerated [175]. Additionally, BBR also has potential therapeutic applications based on antidiabetic effects, such as the increase in insulin receptor expression in type-2 diabetes mellitus, which resulted in a lower blood glucose level [176], as well as the antihyperlipidemic action [177,178] and anticancer properties [179]. Furthermore, in a phase I clinical trial with ulcerative colitis patients, BBR was well-tolerated and decreased the inflammation in colonic tissue [173]. No clinical trials in autoimmune diseases were performed yet.

BBR activated the AMPK pathway on concanavalin-A-induced autoimmune hepatitis (AIH) in mice [127] and affected the JAK/STAT signaling pathway by the reduction in STAT1 and STAT4 phosphorylation, leading to suppressed Th1 differentiation and function in different animal models. Furthermore, BBR also reduced the STAT3 phosphorylation, which, in turn, suppressed Th17 differentiation and function [129,130]. The inhibition of NF-κB activity, a protective effect of BBR, attenuates ROS production and prevents activation of JAK2/NF-κB pathway in diabetic rats [180].

AIH mice show a lower aspartate aminotransferase (AST) and alanine aminotransferase (ALT) level in serum after treatment with 100 or 200 mg/kg BBR and have a reduced TNF-α, IFN-γ, IL-1β, and IL-2 mRNA and protein expression in serum. Moreover, the BBR-treated group had no liver tissue damage [127]. In a rat model of autoimmune neuritis, the experimental model of Guillain–Barré syndrome, an autoimmune disease characterized by inflammatory demyelination and axon damage, the treatment with 20 or 130 mg/kg/day BBR reduced the clinical score. In addition, the treatment suppressed CD4^+^ T-cell proliferation, downregulated the number of IL-10- and TNF-α-positive cells, and reduced serum levels of IgG1 and IgG2a. BBR had no effect on IL-17- and IFN-γ-positive cells in this model [128]. BBR (200 mg/kg/day) also prevented left ventricular dysfunction in experimental autoimmune myocarditis (EAM) in rats and reduced anti-cardiac myosin autoantibodies in serum. Moreover, BBR protected the hearts from pathological changes under EAM and downregulated Th17 and Th1 cells in the hearts of the rats [129]. Additionally, BBR reduced the proinflammatory cytokines IFN-γ, IL-6, and IL-17 in EAE in mice. However, no significant change in IL-4, IL-5, IL-10, and TNF-α levels were observed under treatment. BBR decreased the percentage of CD4^+^ T cells, CD11b^+^ cells, and reduced Th1, Th17, and IL-6 producing CD11b^+^ cells in the spleen and central nervous system of EAE -mice. However, the treatment had no effect of CD8^+^ T cells and B220^+^ B cells [130].

Demethylenebeberine (DMB) is a metabolite of BBR and a component of cortex *Phellodendri chinensis*, a traditional Chinese medicine with antimicrobial, anti-inflammatory, and antidiarrheal effects. It has also been examined in mouse models of inflammatory and autoimmune diseases. In AIH mice, treatment with DMB led to an inhibition of cytokine expression (TNF-α, IL-6, IL-1β, and IFN-γ) on mRNA and protein level. Furthermore, DMB inhibited the phosphorylation of IκB, NF-κB, p68, ERK, JNK, p38MAP, and STAT3 in AIH mice [131]. The DMB treatment (150 or 300 mg/kg) of mice with inflammatory bowel disease resulted in less symptoms, such as reduced loss of body and colon weight, reduced expression of proinflammatory cytokines such as TNF-α and IL-6, as well as lowered nitrogen oxide (NO) production in the colon of these mice. Moreover, it has been reported that DMB decreases the phosphorylation of p65 and IκB in a dose-dependent manner [132]. Therefore, DMB inhibits the NF-κB signaling pathway in various mouse models.

### 3.6. Betulin

The pentacyclic triterpene betulin was first isolated from *Hedyotis hedyotidea* and can be found in many other plants, such as birch bark, mistletoe, and the chaga mushroom *Inonotus obliquus* [181]. *H. hedyotidea* is used in traditional Chinese medicine to treat various diseases, such as gastroenteritis, heatstroke, hepatitis, RA, and herpes zoster. Betulin itself or plant extracts containing betulin have antiviral [182], antifungal, antibacterial, anticarcinogenic [183], and anti-inflammatory, as well immunomodulatory [184] effects. Because of the poor bioavailability of betulin, several semisynthetic ester derivatives with improved solubility and antitumor activity have been synthesized [185].

Betulin reduced inflammation via modulation of TLR4/NF-κB signaling pathway in experimental ulcerative colitis (UC) and kidney injury in septic rats [134,186]. Moreover, betulin showed immune modulating activities by inhibiting activated T cells in autoimmune hepatitis in mice [133]. Additionally, betulin enhanced SIRT1 expression and activated SIRT1/AMPK signaling pathway [187], as well as activating Nrf2 through an AMPK/AKT/Nrf2-dependent mechanism [188,189] in vitro and in vivo.

In acetic-acid-induced UC in rats, betulin (8 mg/kg) attenuated the induced UC, as evidenced by reduced macroscopic scores, serum CRP titer, and lactate dehydrogenase (LDH) activity. In addition, inflammatory cell infiltration, mucosal necrosis, and hemorrhage were reduced in betulin-treated UC rats. Moreover, the production of inflammatory cytokines TNF-α, IL-1β, and IL-6 was reduced in the colon of these rats [134]. Betulin (10 or 20 mg/kg) also decreased serum levels of TNF-α, IFN-γ, and IL-6 in AIH in mice, ameliorated liver injury, and inhibited natural killer T cells, as well as conventional T-cell activation. No significant changes were observed in serum levels of IL-2, IL-4, IL-10, and IL-17 in AIH mice. The authors described betulin as a treatment option in T-cell-dependent autoimmune diseases [133].

Two clinical phase 3 trials were performed with betulin gel on skin wounds [190] and partial burns [191]. Both showed that betulin gel is well-tolerated and safe.

### 3.7. Curvularin (Cur)

The fungal macrocyclic lactone curvularin (Cur) was first isolated from the imperfect fungi *Penicillium gilmanii* and *P. baradicum* [192,193].

Cur inhibited expression of inflammatory mediators (TNF-α, IL-1β, IL-6) and metalloprotease (MMP-2, MMP-3, a disintegrin and metalloproteinase with thrombospondin motifs 5 (ADAMTS-5)) in vitro in the context of inflammation in intervertebral disc degeneration and was described as a potential alternative to steroids in this study [194]. Additionally, Cur and derivatives of Cur showed an anti-inflammatory effect via inhibition of NO and prostaglandin E2 overproduction in RAW 264.7 macrophages [135]. The (S)-enatiomer of curvularin (S-Cur) has also been described as an inhibitor of proinflammatory gene expression and activated JAK2 and STAT1 in vitro, indicating that S-Cur inhibited STAT1-dependent gene expression [195]. Furthermore, S-Cur also inhibited the binding of the activated transcription factors Smad2 and Smad3 to the DNA and act antagonistically on the effects of TGF-β in vitro [136].

A study by Schmidt et al. [137] showed the effect of S-Cur treatment in CIA mice, a model of RA. S-Cur treatment with 10 mg/kg inhibited the protein expression of the chemokines MCP-1, CCL17, macrophage inflammatory protein (MIP)-1α, MIP-1β, MIP-2, and CXCL12, as well as the cytokines TNF-α, IFN-γ, IL-6, IL-10, and IL-12p70 in CIA mice, whereas the treatment control with 5 mg/kg of the glucocorticoid dexamethason (DEX) shows no or lower reduction in IFN-γ and IL-1β. Schmidt et al. identified in microarray analyses 20 genes, significantly up- (e.g., neurogene locus-notch-homolog-protein 2 (NOTCH2) and forkhead box C1 (Foxc1)) or downregulated (e.g., STAT1, cyclic adenosine monophosphate (cAMP) and S100 calcium-binding protein A8 (S100A8)), involved in signal transduction and immune defense mechanism in S-Cur-treated CIA mice. They were partially less regulated in the DEX-treated group. Additionally, S-Cur reduced in vitro the mRNA expression of IL-8, MCP-1, TNF-α, and COX2 in human chondrocyte cell line C28/I2, which do not respond to glucocorticoids [196]. Taken together, in CIA mice S-Cur treatment reduced the inflammatory process similar to the established glucocorticoid DEX and is also effective in a glucocorticoid-resistant cell model [137].

So far, there are no clinical trials on either Cur or S-Cur.

### 3.8. Oxacyclododecindione (OXA)

Oxacyclododecindione (OXA) is a macrocyclic lactone which was isolated from the imperfect fungus *Exserohilum rostratum* as a potent inhibitor of IL-4 signaling [197]. OXA belongs to the class of fungal 12-membered macrolactones of the curvularin type, for which mainly antibacterial, antifungal, and antitumor activities have been recently described [198,199]. Initial in vitro studies revealed that OXA inhibited IL-4 signaling by blocking the binding of the activated transcription factor STAT6 to the DNA without affecting tyrosine phosphorylation [188]. In a following study, it was reported that OXA antagonized TGF-β-dependent cellular effects, including reporter gene activity, DNA binding of the activated transcription factors Smad2 and Smad3, expression of TGF-β induced genes related to angiogenesis and metastasis and blocked capillary-like tube formation of the highly invasive breast cancer cell line MDA-MB-231 [186]. Two derivatives of OXA, 14-dechloro-14-deoxy-OXA and 14-deocy-OXA, isolated from the same fungus were found to exhibit antifibrotic activities by inhibiting TGF-β-induced connective tissue growth factor (CTGF) promotor activity, blocking CTGF mRNA levels, CTGF protein expression, and tube formation in vitro in a dose-dependent manner [200]. OXA and 14-deoxy-oxacyclododecindione exerted a highly potent inhibition of TGF-β signaling with IC_50_ values in the nanomolar range.

Henke et al. [138] demonstrated the anti-inflammatory effects of OXA in the SLE model of MRL/lpr mice. OXA treatment (1 mg/kg) reduced the mRNA expression of IFN-γ, IL-6, and TNF-α, as well as the TNF-α, secreted phosphoprotein 1 (SPP1), and S100A8 protein levels. Furthermore, OXA treatment led to a significant reduction in chemokines (CCL2, CCL4, CCL5, CXCL9, and CXCL12) and cytokines (IFN-γ, TNF-α, IL-16, IL-17, IL-23, IL-27, and colony-stimulating factor 1 (CSF1)), involved in pathogenesis of lupus glomerulonephritis, in kidneys of OXA-treated SLE mice, as well as a reduction in kidney damage and immune cell infiltration (T cells, monocytes, macrophages, and B cells) into the kidneys. Moreover, OXA improved kidney disease as measured by reduction in proteinuria, renal IgG deposition, and dsDNA antibodies. In addition, OXA reduced the development of fibrosis in kidney. In summary, OXA ameliorated glomerulonephritis of SLE mice [138]. An OXA-mediated inhibition of the p38-MAPK pathway seems to contribute to the anti-inflammatory effect (unpublished data). Due to its dual effect on inflammation and fibrosis, OXA may also be an interesting drug candidate for the treatment of tubulointerstitial-derived chronic kidney diseases where tubular injury and interstitial fibrosis are important for disease progression [201]. Interesting is the low dosage of 1 mg/kg needed for this effect; for comparison, 5 mg/kg DEX was necessary in this study [138] to obtain a similar effect. It should be noted that the other natural products used in the above-mentioned inflammatory animal models were applied in concentrations of 8–20 mg/kg for betulin [133,134], 50–75 mg/kg for DHA [122,123], or 20–160 mg/kg for HES [40,41].

There are currently no clinical trials on the lactone OXA.

## 4. Conclusions

A number of natural products, such as cyclosporine, are used in the clinic as immunosuppressants successfully. This demonstrates the importance of fungi, herbs, and microorganisms as sources for identification of new therapeutic structures. Several new interesting candidates with anti-inflammatory and immunosuppressive function have been identified over the past years. A common mode of action of those compounds seems to be inhibition of NF-κB and JAK/STAT signaling pathways also addressed by a number of target-specific drugs, such as TNF-α or JAK inhibitors. In addition, most of the natural products seem to have the ability to reduce ROS levels, which contributes to their anti-inflammatory effect and constitutes a difference to the commonly used drugs in the clinic. In addition, natural products which possess anti-inflammatory and antifibrotic effects in parallel, such as the macrocyclic lactone OXA, may also be interesting for the treatment of chronic kidney diseases. All substances have more or less in common that they have a poor or limited bioavailability in the originally isolated form. This makes it necessary to chemically modify the substances to enable forms of simple administration without simultaneously losing the efficacy of the substance. In many cases, this is probably also the reason for the lack of clinical studies to date, despite the availability of a large number of promising in vitro and in vivo data.

## Figures and Tables

**Table 1:** Natural products tested in animal models and in clinical trials of autoimmune diseases.

Compound	Structures	Class	Models			Dosage	References
			In Vitro	In Vivo	Clinical Trials		
Curcumin	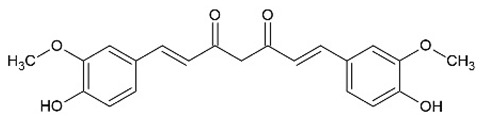	Diarylheptanoid	Murine macrophages cell line RAW 264.7	Acute kidney injury model: cisplatin-induced mice			[8]
			Breast cancer cell line MDA-MB-231			25 µM	[9]
				Systemic lupus erythematosus (SLE) model: NZBWF1 mice		500 mg/kg/d	[10]
			Murine macrophages cell line RAW 264.7	Rheumatoid arthritis (RA) model: collagen-induced arthritis (CIA) rats			[11]
				Inflammatory bowel disease model: DSS-induced mice		50 mg/kg	[12]
					RA	250 or 500 mg/twice a day	[13,14]
					Oral lichen planus	80 mg/d	[15]
Resveratrol	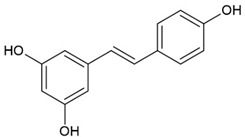	Polyphenolic Phytoalexin		Autoimmune myocarditis model: Cardiay myosin immunized rats		50 mg/kg	[16]
				RA model: BIIC-induced rats		200 or 400 mg/kg	[17]
				SLE model: pristine-induced mouse		25 or 50 mg/kg	[18]
					RA	1 g/d	[19]
Quercetin	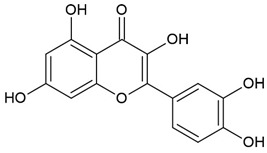	Flavonoid		RA model: Zymosan-induced mice			[20]
				RA model: CIA mice		30 or 150 mg/kg	[21,22]
				SLE model: chronic graft vs. host disease (cGVHD) mice		80 mg/kg	[23]
				SLE model: pristine-induced mouse			[24]
					RA	500 mg/d	[25]
Sinomenine	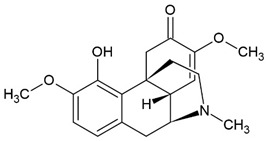	Alkaloid	Murine macrophages cell line RAW 264.7	RA model: CIA mice		50 µg/mL; 50 mg/kg/d	[26]
			human monocyte-derived dendritic cells				[27]
				Multiple sclerosis (MS) model: experimental autoimmune encephalomyelitis (EAE) rats		50, 100 or 200 mg/kg/d	[28]
					RA	60–120 mg/d	[26,29]
Baicalein	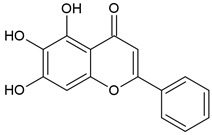	Flavonoid		RA model: CIA mice		20 mg/kg	[30]
Baicalin	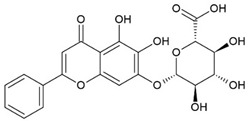	Flavonoid		Colitis model: DSS-induced mice		20 mg/kg	[30]
			Murine macrophages cell line RAW 264.7	Colitis model: TNBS-induced colitis rats		1.25–5 mg/mL/d lavage	[31]
				MS model: EAE rats		100 mg/kg/d	[32]
					RA patients with coronary artery disease	500 mg/d	[33]
Paeoniflonrin	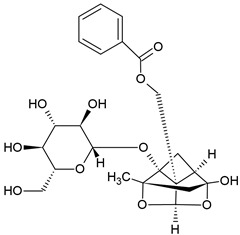	Monoterpene Glucoside		Colitis model: TNBS-induced colitis mice		15, 30 or 45 mg/kg	[34]
			RA-fivroblast-like synoviocytes (FLSs)			25, 50 or 100 µM	[35]
					RA		[36]
Hesperidin	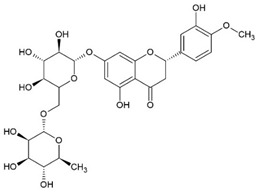	Flavonoid Glycoside	Murine macrophages cell line RAW 264.7				[37]
				Asthma model: OVA-induced asthma mice		1, 5, 10 or 30 mg/kg	[38,39]
				RA model: antigen-induced arthritis (AIA) mice		20 mg/kg/d	[40]
				RA model: adjuvant arthritis (AA) rats		80 or 160 mg/kg	[41]
					RA	3 g/d	[42]

**Table 2:** Natural products tested in animal models of autoimmune diseases.

Compound	Structures	Class	Model		Dosage	References
			In Vitro	In Vivo		
Celastrol	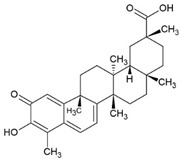	Triterpenoid		Rheumatoid arthritis (RA) model: adjuvant arthritis (AA) rats	1 mg/kg/d	[113]
				RA model: antigen-induced arthritis (AIA) mice	3 g/kg/d	[114]
			2D and 3D model of psoriasis		3, 10, 30 or 90 ng/mL	[115]
Glycyrrhizin	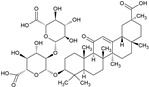	Triterpenoid Saponin		RA model: collagen-induced arthritis (CIA) rats		[116]
Artemisinin	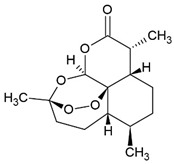	Sesquiterpene Lactones		RA model: CIA mice	5–20 mg/kg/d	[117]
				Systemic lupus erythematosus (SLE) model: MRL/lpr mice		[118]
			human umbilical vein endothelial cells (HUVECs)		5 or 20 µM	[119]
			peripheral blood mononuclear cells (PBMCs) from SLE patients		5 or 20 µM	[119]
			Spleen cells from MRL/lpr mice		0.1–10 µM	[120]
				SLE model: pristine-induced mouse	100 mg/kg/d	[121]
				Acute lung injury (ALI) model: lipopolysacchaide-induced ALI mice	75 mg/kg	[122]
				Asthma model: OVA-induced asthma mice	50 mg/kg	[123]
Sophocarpine	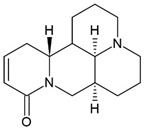	Alkaloid	Fibroblast-like synoviocytes (FLSs)	RA model: CIA mice		[124]
				SLE model: MRL/lpr mice	100 mg/kg/d	[125]
				Asthma model: OVA-induced asthma mice		[126]
Berberine	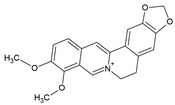	Alkaloid		Autoimmune hepatitis (AIH) model: Concanavalin-A-induced AIH mice	100 or 200 mg/kg	[127]
				Guillain-Narré syndrome model: experimental autoimmune neuritis rats	20 or 130 mg/kg/d	[128]
				Myocarditis model: experimental autoimmune myocarditis (EAM) rats	200 mg/kg/d BBR	[129]
Demethylenebeberine	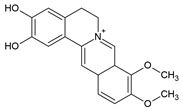			Multiple sclerosis (MS) model: experimental autoimmune encephalomyelitis (EAE) mice	200 mg/kg/d	[130]
				AIH model: Concanavalin-A-induced AIH mice		[131]
				Inflammatory bowel disease model: dextran-sulfate-sodium-induced inflammatory colitis mice	150 or 300 mg/kg	[132]
Betulin	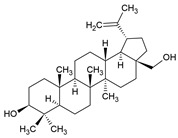	Pentacyclic Triterpen		AIH model: Concanavalin-A-induced AIH mice	10 or 20 mg/kg	[133]
				Ulcerative colitis (UC) model: acetic-acid-induced UC rats	8 mg/kg	[134]
Curvularin	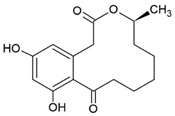	Macrocyclic Lactone	Murine macrophages cell line RAW 264.7			[135]
			Hepatocarcinoma cell line HepG2 and breast carcinoma cell line MDA-MB-231		17.1–171 µM	[136]
			human chondrocyte cell line C28/I2	RA model: CIA mice	10 mg/kg	[137]
Oxacyclododecindione	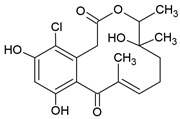	Macrocyclic Lactone	Hepatocarcinoma cell line HepG2 and breast carcinoma cell line MDA-MB-231		86–1350 nM	[136]
				SLE model: MRL/lpr mice	1 mg/kg	[138]

## Data Availability

Data sharing not applicable.
